# Identification of Under-Detected Periodicity in Time-Series Microarray Data by Using Empirical Mode Decomposition

**DOI:** 10.1371/journal.pone.0111719

**Published:** 2014-11-05

**Authors:** Chaang-Ray Chen, Wun-Yi Shu, Cheng-Wei Chang, Ian C. Hsu

**Affiliations:** 1 Department of Biomedical Engineering and Environmental Sciences, National Tsing Hua University, Hsinchu, Taiwan; 2 Institute of Statistics, National Tsing Hua University, Hsinchu, Taiwan; Queen’s University Belfast, United Kingdom

## Abstract

Detecting periodicity signals from time-series microarray data is commonly used to facilitate the understanding of the critical roles and underlying mechanisms of regulatory transcriptomes. However, time-series microarray data are noisy. How the temporal data structure affects the performance of periodicity detection has remained elusive. We present a novel method based on empirical mode decomposition (EMD) to examine this effect. We applied EMD to a yeast microarray dataset and extracted a series of intrinsic mode function (IMF) oscillations from the time-series data. Our analysis indicated that many periodically expressed genes might have been under-detected in the original analysis because of interference between decomposed IMF oscillations. By validating a protein complex coexpression analysis, we revealed that 56 genes were newly determined as periodic. We demonstrated that EMD can be used incorporating with existing periodicity detection methods to improve their performance. This approach can be applied to other time-series microarray studies.

## Introduction

Microarray technologies allow genome-wide gene expression to be measured. Although microarray data are noisy, attempts have been made to elucidate the sources of this noise and to reduce its impact [1]–[3]. Time-series microarray studies have commonly been used to monitor gene activity at the transcriptomic level over an evolving period [4]–[7]. Confounding experimental issues, including synchronization loss [8], [9], RNA degradation [10], [11], and weakly expressed genes [12], challenge the analysis of time-series microarrays. The most prominent example of the effects of these confounding issues is the controversy surrounding the poor overlap between cell-cycle-regulated genes identified using a collection of methods [13].

Recent surveys of the methods used for time-series periodicity detection showed that each method had different strengths and limitations [14]–[17]. This lack of a universal method for comprehensively examining oscillatory behavior highlights limitations of periodicity detection for microarray time-series data. Oscillations within a time series are detected based on the characteristics of the data structure. The algorithms used to interpret the data structure vary by application [16], [17]. Whether and how oscillations affect the performance of periodicity detection is poorly understood. Tools that can assess how oscillations directly contribute to the detected periodicity are urgently required. Therefore, we propose a new framework in this paper to address these problems.

**Figure 1:**
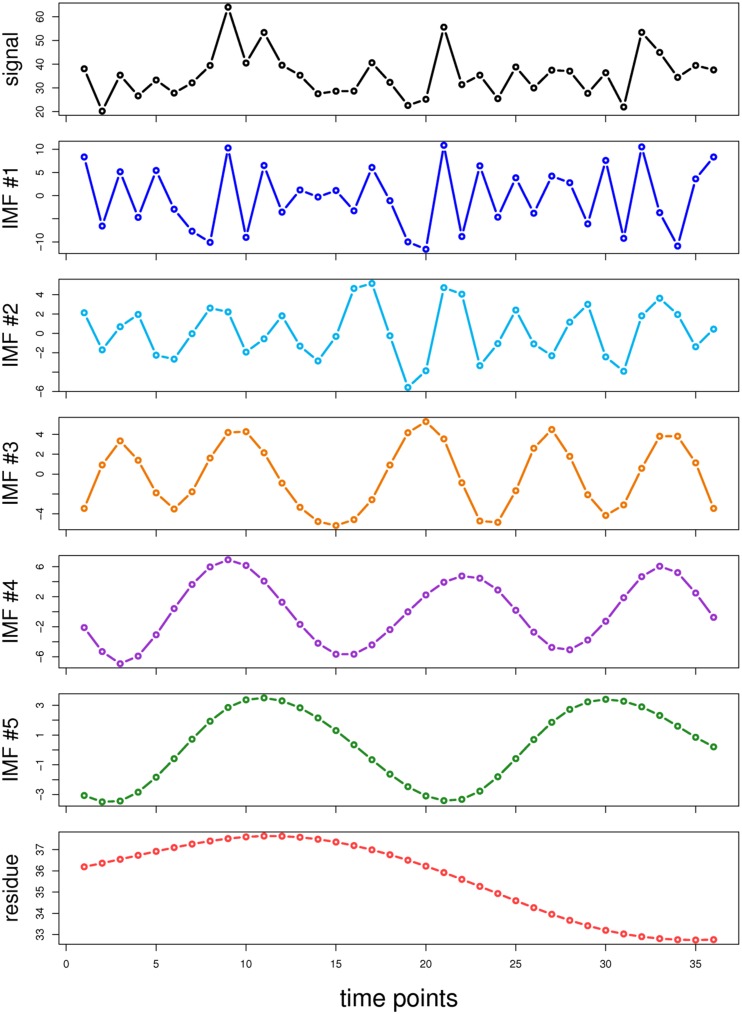
Demonstration of EMD.

Empirical mode decomposition (EMD) is an adaptive time-frequency data analysis method that decomposes any time series into a collection of components called intrinsic mode functions (IMFs) [18]. The extracted IMFs characterize a set of oscillations from the highest to the lowest frequencies in a stepwise fashion (Figure 1). The objective of EMD is to iteratively identify an oscillation or IMF embedded in a time series at a local time scale by performing a sifting process. The sifting process involves the following steps: (i) identifying the local maxima and minima from a time series; (ii) connecting the upper and lower envelopes of the data by interpolating between the local maxima and minima, respectively, using a natural cubic spline; (iii) extracting the first prototype IMF by subtracting the mean envelope value from the targeted time series; and (iv) repeating procedures (i) to (iii) until no further IMFs can be extracted. These IMFs satisfy two conditions: (i) the number of extrema and the number of zero-crossings differ by one, and (ii) the mean value of the upper and lower envelopes is zero. EMD has been widely used to delineate trends and to implement detrending operations during data analysis [19], [20], and it has been successfully used for a broad range of science and engineering applications [21]–[27]. For example, in a transcriptomic analysis, Wang et al. proposed a novel EMD-based method for clustering microarray data [28].

In this study, we extended the application of EMD to the analysis of time-series microarray studies. Recently, Tu et al. demonstrated that approximately 52% of a genome was periodically expressed in yeast metabolic cycles (YMC) by taking samples during three consecutive intervals of 300 min every 25 min, producing data at 36 time points [29]. We applied EMD to the YMC dataset to extract IMFs or oscillations and to assess the impact of IMF oscillations on periodicity detection. We observed that Tu et al. might have under-detected 1394 genes because of higher levels of IMF oscillations. We constructed a panel of 279 benchmark genes to validate the under-detected genes by using a protein complex coexpression analysis. Tu et al. identified 211 (76%) genes, but our results suggested that 267 (96%) genes, including 56 newly determined genes, were periodically expressed. Most of the under-detected genes were mainly associated with ribosome biogenesis and RNA processing.

Our results demonstrated that EMD is a powerful tool that can be used to compensate for the impact of the complexity of oscillation and to improve the performance of existing periodicity detection methods. The framework proposed in this paper can be applied to other time-series microarray studies.

## Results

### Reanalysis of the yeast metabolic cycle dataset

**Figure 2:**
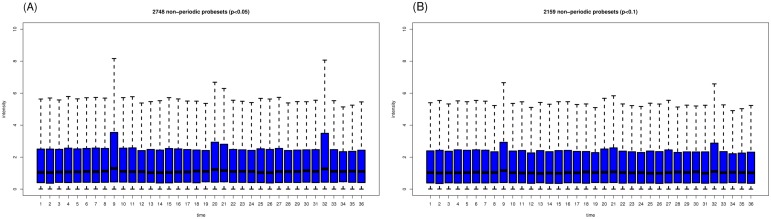
Boxplots of the expression profiles of non-periodic probesets with two cut-off criteria: (A) p<0.05 and (B) p<0.1.

We reanalyzed the YMC dataset by using a slightly modified data preprocessing protocol (see Methods) and applied the same cut-off criterion (p<0.05) for periodicity detection. Of the 6537 unique probesets, 3789 (57.96%) were periodically expressed. The average expression level of the periodic probesets was approximately 1.7-fold higher than the average expression level of the 2748 (42.03%) non-periodic probesets. Figure 2A indicates that many of the non-periodic probesets tended to peak at time points 9, 20 or 21, and 32. To determine whether the differences in the expression of these probesets might be too close to the cut-off value, we reanalyzed the YMC dataset with a less stringent criterion (p<0.1). We observed that many of the 2159 (33.03%) non-periodic probesets still tended to peak at time points 9 and 32 (Figure 2B).

### Non-periodic probesets exhibit a higher level of IMF oscillations

**Figure 3:**
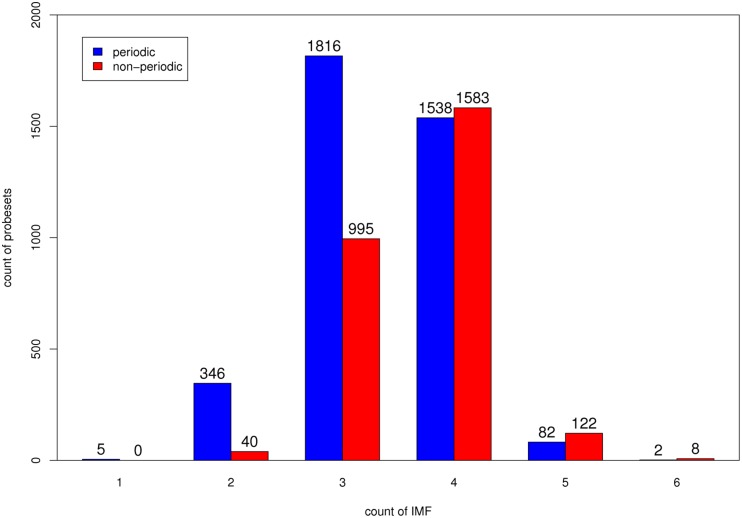
Impact of the IMF. Distribution of the number of extracted IMFs for 6537 YMC probesets.

**Figure 4:**
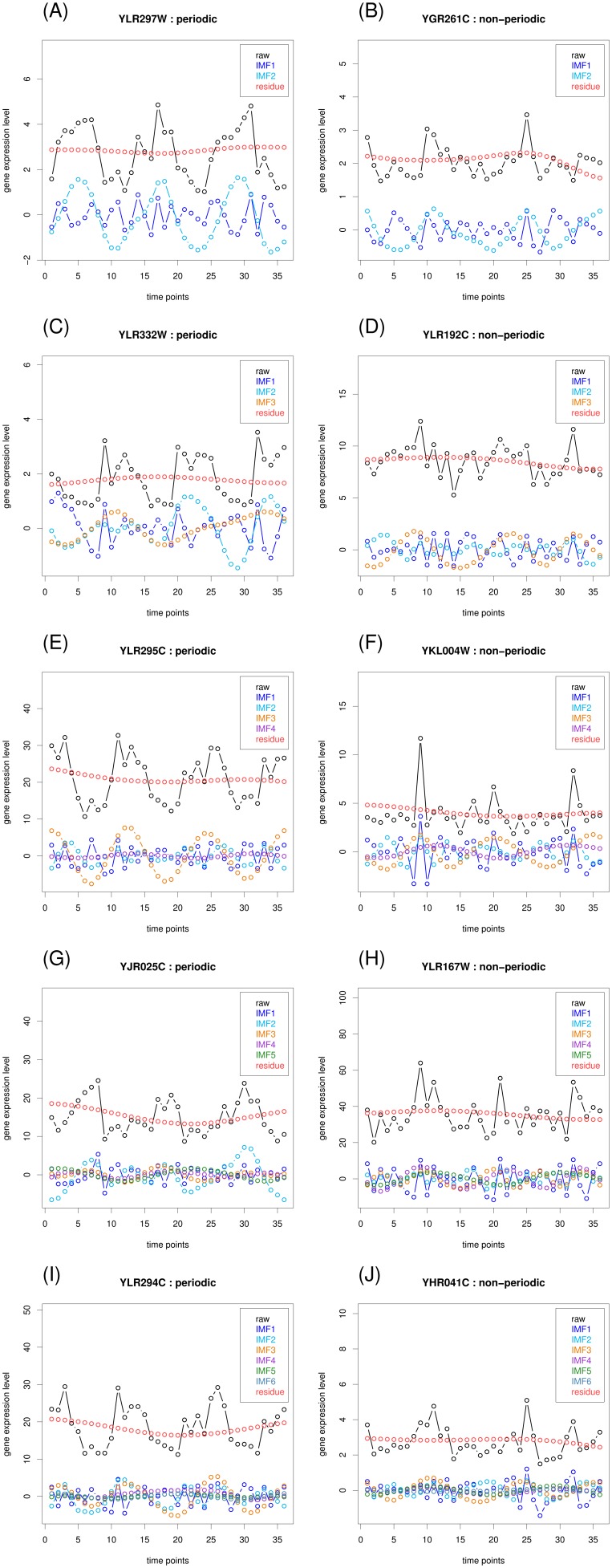
Examples of expression profiles for periodic and non-periodic probesets that were decomposed into two to six IMFs. The left column presents the periodic probesets. The right column presents the non-periodic probesets.

To improve understanding of the characteristics of the temporal structure of the YMC dataset, we analyzed the dataset by using EMD. The expression level of a probeset can be decomposed into a series of oscillations (IMFs) and a residual trend (Figure 1). In general, the maximal number of extracted IMFs is proportional to the logarithm (base 2) of the data length [18]. The 36 time points of the YMC probesets were decomposed into as many as six IMFs. We divided all of the probesets into six groups according to the number of decomposed IMFs and compared the distributions of the periodic and non-periodic probesets. The results demonstrated that more IMFs were decomposed from non-periodic probesets than from periodic probesets (Figure 3), indicating that a higher level of oscillations was embedded in the non-periodic probesets. Five of the 3789 periodic probesets consisted of a single IMF, from which a sinusoid was extracted, regardless of the expression level (Figure S1). Periodic probesets consisting of two to six components reflect that some dominant IMFs might have contributed to the detected periodicity (Figure 4; left column). However, some dominant IMFs of the non-periodic probesets might have contributed to periodicity, but with interference from other components (Figure 4; right column).

### Identification of under-detected periodicity

To decipher how IMFs contribute to the performance of periodicity analysis, we developed a reverse-engineering approach involving an IMF-based interpolation of the original time series. All of the possible combinations of IMFs for each probeset were reconstructed as a new time series. Based on the number of extracted IMFs of a probeset n, a total of n^2^-1 combinatorial IMFs could be interpolated. The algorithm described by Tu et al. was then used to determine whether the newly reconstructed time series were periodic. This approach enabled us to search all the intrinsic patterns derived from the original time series.

**Figure 5:**
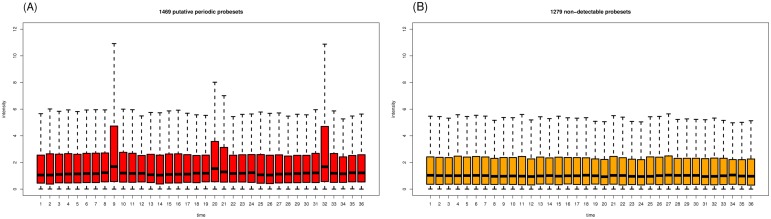
Boxplots of the expression profiles for (A) 1469 PP and (B) 1279 ND probesets.

**Figure 6:**
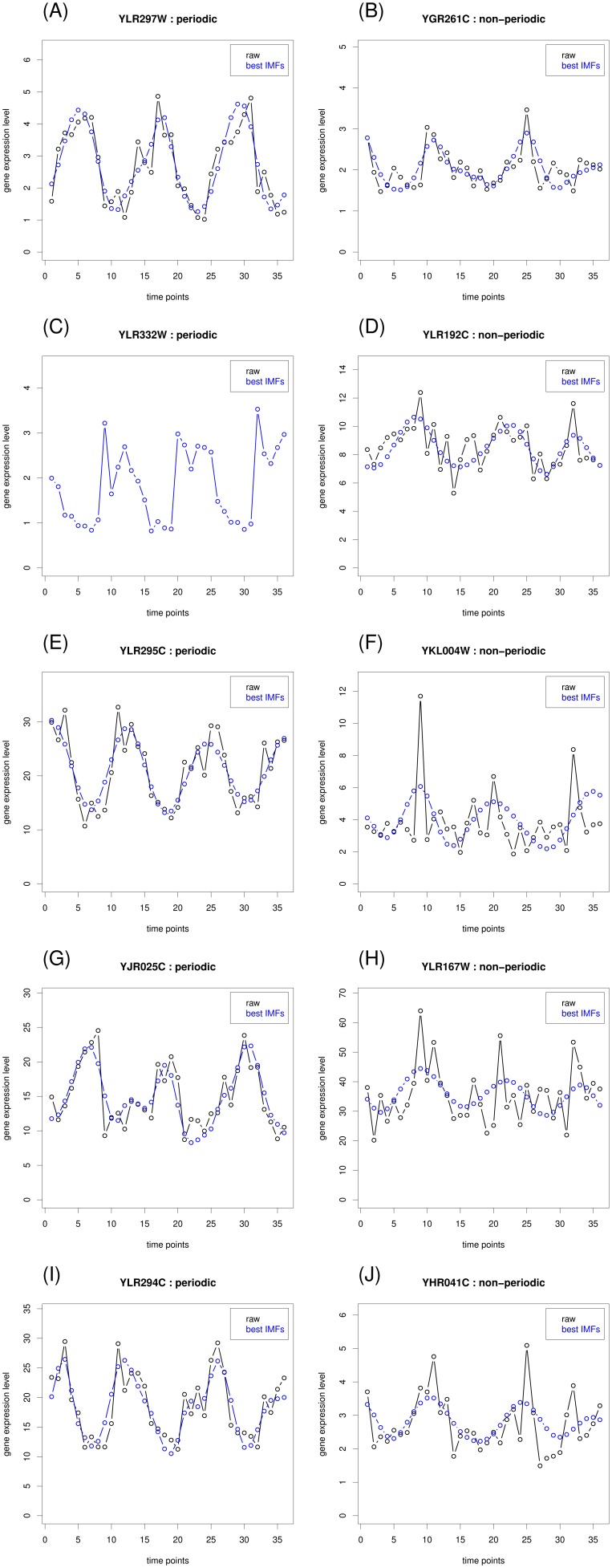
Expression profiles for the optimal interpolated combinatorial IMFs, which correspond to the examples in Figure 4. The left column presents the periodic probesets. The right column presents the non-periodic probesets.

We compared the optimal combinatorial IMF (with the minimal p value) with the original time series to identify the IMF(s) that directly contributed to or interfered with the detected periodicity. Consequently, the 2748 non-periodic probesets were further divided into two subgroups: non-detectable (ND) and putative periodic (PP) probesets. Of the 1279 ND probesets (46.54%), none of the reconstructed combinatorial IMFs were periodic. In agreement with Tu et al., these probesets were non-periodically expressed. However, we observed that the optimal combinatorial IMFs of the 1469 PP probesets (53.45%) were periodic. The average expression level of the PP probesets was similar to the average expression level of the ND probesets, but the PP probesets peaked at time points 9, 20 or 21, and 32 (Figure 5). This suggested that the periodicity of these PP probesets might have been under-detected because of interference from other IMFs. The optimal combinatorial IMFs corresponding to the examples in Figure 4 are presented in Figure 6. Five example genes in Figure 6 (right column) were under-detected by Tu et. al, but were identified by our EMD approach.

### Validation of under-detected genes

We constructed a panel of benchmark genes to validate the under-detected probesets by using a coexpression analysis. A recent study reported that the expression of more than 85% of yeast genes was affected by nine large, highly coexpressed protein complexes totaling to 303 genes [30]. Liu et al. [30] conducted a coexpression analysis by calculating the correlations between the expression levels of genes that encode elements of protein complexes contained in the MIPS catalog [31] based on four public microarray datasets (approximately 600 experimental conditions). To exclude the possibility that the identification of these highly coexpressed gene pairs might have been biased by the limited size of the dataset, we conducted a similar coexpression analysis (see Methods) and integrated the large SPELL compendium [32]. This approach enabled us to examine more coexpression patterns of gene pairs encoding subunits of the same protein complex.

In the YMC dataset, 279 genes were associated with these highly coexpressed protein complexes, suggesting that these genes are periodically expressed. In other words, these genes can support the discovery of under-detected periodicity. Whereas Tu et al. identified 211 (76%) periodic genes, our coexpression analysis suggested that 56 genes were under-detected. This result demonstrated that performing EMD substantially improved the detection of periodic genes, leading to the identification of 267 (96%) genes (Table 1). Over 80% of the gene pairs from nine large protein complexes (except for the rRNA splicing complex that consists of 24 proteins) were coexpressed with confidence levels of ≥95% (Table 1). This result demonstrated that a gene tends to be periodically expressed, similar to other highly correlated genes in the same protein complex.

**Table 1:** Summary of nine highly coexpressed MIPS protein complexes.

MIPS protein complex (ID)	#.	#.	Periodic genes	Putative periodic genes	Total gene pairs	Coexpressed gene pairs	Ratio
	proteins	YMC genes					
mitochondrial ribosomal large subunit (500.60.10)	44	44	44	0	946	932	0.985
mitochondrial ribosomal small subunit (500.60.20)	31	31	30	0	465	391	0.841
cytoplasmic ribosomal large subunit (500.40.10)	81	66	55	11	2145	2132	0.994
cytoplasmic ribosomal small subunit (500.40.20)	57	49	36	11	1176	1176	1
19/22S regulator (360.10.20)	18	18	7	7	153	151	0.987
20S proteasome (360.10.10)	15	15	8	4	105	105	1
F0/F1 ATP synthase, complex V (420.50)	18	18	12	5	153	142	0.928
rRNA splicing (440.30.20)	24	24	12	12	276	126	0.457
H+-transporting ATPase, vacuolar (220)	15	14	7	4	91	73	0.802

**Figure 7:**
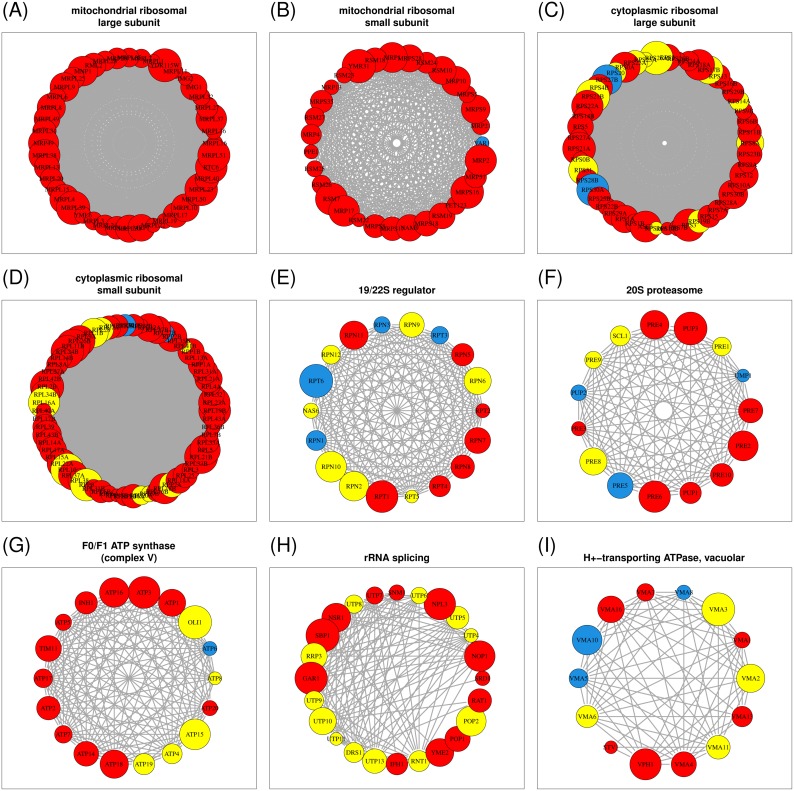
Visualization of networks of nine highly coexpressed MIPS protein complexes. A colored node represents a gene and the radius of the node is proportional to the intensity or gene expression level. An edge between two nodes indicates that a gene pair is coexpressed. A red node reflects a periodically expressed gene identified by both Tu et al. and EMD. A yellow node indicates a periodically expressed gene identified by EMD, but not by Tu et al. A blue node indicates a periodically expressed gene identified by neither Tu et al. nor EMD.

Coexpressed protein complexes are visualized using network graphs, in which a node indicates a gene and an undirected edge between nodes indicates a gene pair that is coexpressed (see Methods). As shown in Figure 7, the expression levels of these protein complex components were highly correlated; the more edges between nodes (genes), the higher the level of coexpression of genes encoding elements of a protein complex. The best examples of coexpression were provided by mitochondrial ribosomal complexes (Figures 7A and 7B). Tu et al. identified all the periodically expressed genes, except for one (Table 1). The expression levels of nearly all the gene pairs of cytoplasmic ribosomal complexes were highly correlated, but many of these genes were identified as non-periodic in the Tu et al. analysis (Figures 7C and 7D). The discovery of PP genes in these complexes might indicate that under-detected periodicity led to the discrepancy between the results of the original analysis and the coexpression analysis. The subcomponents of proteasome complexes were highly coexpressed. All the gene pairs encoding subcomponents of the 20S proteasome were correlated, and only two of the 153 gene pairs of the 19/22S regulator were not correlated (Table 1). We identified seven and four under-detected genes from the 19/22S regulator and the 20S proteasome complex, respectively (Figures 7E and 7F).

Four of the five protein complexes (Complexes II, III, IV, and V) involved in oxidative phosphorylation were included in the MIPS database [31]. Only Complex V (FO/F1 ATP synthase, 18 proteins) was identified by Liu et al. as a strongly coexpressed protein complex because they focused on large complexes (≥15 proteins). Our analysis indicated that Complexes II, III, and IV were also highly coexpressed (Table S1). Five genes from Complex V were newly determined to be periodic (Figure 7G). With the exception of one gene, all the genes of Complexes II, III, and IV were also identified as periodically expressed (Figure S2). Similarly, all of the genes of the rRNA splicing complex reflected periodicity (Figure 7H).

### Most under-detected genes were associated with the oxidative phase of the metabolic cycle

In Tu et al., YMC transcriptome profiling revealed three major metabolic phases: oxidative (OX, 1023 genes), reductive-building (RB, 977 genes), and reductive-charging (RC, 1510 genes) phases. We examined the most recently updated version (April 2014) of the gene annotations for the YMC dataset, and approximately 4% of the periodic genes were excluded because of discrepancies between the gene annotation versions. The revised numbers of genes in the OX, RB, and RC clusters were 986, 946, and 1439, respectively.

**Figure 8:**
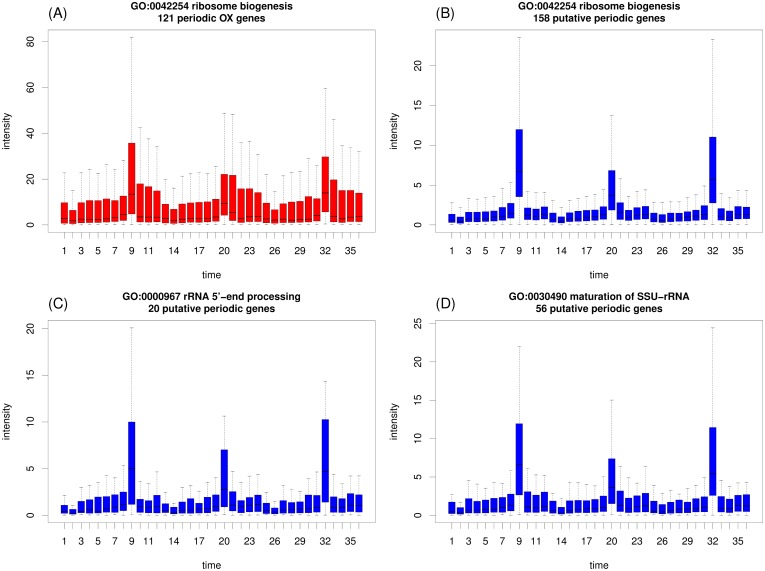
Boxplots of expression profiles for genes associated with enriched GO terms.

To gain a comprehensive understanding of the functional roles of the 1469 PP probesets, a gene ontology (GO) enrichment analysis [33] was conducted. The enriched GO terms (in the biological process (BP) category) of the PP probesets were compared with those of the OX, RB, and RC clusters (Table S2). The results clearly indicated that these clusters comprised distinct subclasses of genes associated with various biological processes. Nearly all of the common GO terms for the PP probesets were shared with the OX cluster (Table S2). Confirming the results reported by Tu et al., the functions associated with these GO terms were primarily related to nucleic acid metabolic processes, ribosome biogenesis, and rRNA processing. Table S2 shows a complete list of enriched GO terms. Furthermore, many PP genes in the OX cluster peaked periodically and abruptly during the cycles. Tu et al. suggested that these OX genes might play critical regulatory roles in ensuring tight coupling between transcription and metabolic cycles. As shown in Figure 8 (top row), a typical example of a common GO term is ribosome biogenesis (GO:0042254). The average expression level of 121 OX genes was approximately 2.5-fold higher than the average expression level of 158 PP genes, indicating that low expression levels might contribute to the under-detection of periodicity. Moreover, the distribution of the number of IMFs demonstrated that the PP genes were decomposed into more IMFs than the OX genes (Figure S3), suggesting that interference between IMF oscillations might also lead to the under-detection of low-intensity periodicity.

Although many of the enriched GO terms were specific to PP probesets, most of the terms were similar to terms common to OX genes, which are associated with rRNA, RNA, and ribosomes (Table S2). The genes of many rRNA- and ribosome-associated PP-specific GO terms peaked abruptly at a single time interval in the cycle (Figure 8, bottom row), indicating that these weakly expressed genes are associated with the genes of the OX cluster.

## Discussion

In recent decades, intensive efforts have been exerted to compare existing periodicity detection methods, but each method has different strengths and limitations [14]–[17]. A recent survey of four types of mathematical methods for detecting periodic signals recommended selecting a method based on noise type, sampling rate, and curve shape [17]. This comparative study addressed both the strengths and limitations of existing methods. However, the possibility of adaptively improving the limitations of existing methods has not been explored. In this paper, we propose a proof-of-principle framework using EMD to address this challenge. We applied this approach to the YMC dataset involving three steps.

First, we demonstrated that the data structure can be extracted using a series of IMFs without any data shape assumptions. These IMFs extend the dimensions of the original time series and can provide additional information on the empirical data structure. This process enabled us to comprehensively examine and visualize oscillations within the original time series (Figure 1).

Second, a universal benchmark was proposed to evaluate the performance of existing periodicity detection methods. We defined levels of oscillation within a time series by using the number of extracted IMFs. This simple quantized measure can divide time-series microarray data into groups based on the results of an existing periodicity detection method across a discrete spectrum of oscillations (Figure 3).

Third, we analyzed the intrinsic periodicity of all the possible combinatorial IMFs to identify the IMFs that directly contributed to or affected the detected periodicity. These combinatorial IMFs can be used to examine the effects of superposed IMFs and to address the limitations of existing periodicity detection methods. Moreover, this reverse-engineering approach enabled us to compensate for the effect of IMFs by identifying the periodicity of the optimal combinatorial IMF instead of the periodicity of the original time series.

Our EMD-based framework is a simple tool that can be used for benchmarking and for leveraging periodicity analyses beyond their limitations. In this YMC study, we observed that 1469 probesets representing 1394 genes might have been under-detected by Tu et al. (Table S3). Combined with the 3789 probesets identified in the original analysis, the total number of periodic probesets might be as large as 5258. Detecting the same number of periodic probesets without EMD resulted in a p value of 0.31. Moreover, if the 5258 probesets were identically matched without EMD, the periodicity analysis became a process of random selection (p<0.95).

Recently, Park et al. [34] proposed a framework for clustering dynamic protein complex networks by integrating the same YMC dataset, protein complex coexpression, and protein-protein interaction maps. They predicted 31 dynamic protein complex networks using approximately 3500 periodically expressed genes identified by Tu et al. and compared these networks to two references of manually curated protein complex databases. Of the 31 predicted complexes, 6, 11, and 14 complexes exhibited good, moderate, and poor overlap with the references, respectively. They also tested the effects of data filtering by using all the YMC genes, but the overlap with the protein complex references was similar for the filtered network. We propose that analyzing the unfiltered network by using our under-detected genes, instead of all the non-periodic YMC genes, might improve the overlap between the prediction and references. Moreover, our findings suggested that up to 80% of yeast protein-coding genes might be periodically expressed during the metabolic cycle. We suggest that these under-detected genes might provide useful resources for investigating potential regulatory targets in the yeast metabolic cycle.

Our reanalysis of the YMC dataset revealed that the under-detection problem was not case-specific to the algorithm described by Tu et al. We also applied a different periodicity detection algorithm [35] to the YMC dataset. We found that the expression profiles of many non-periodic probesets also tended to peak at time points 9, 20 or 21, and 32 (Figure S4A). We then reanalyzed the dataset with EMD, resulting in the consistent division of the non-periodic probesets into two subgroups (Figures S4B and S4C). In total, 2179 probesets might have been under-detected.

In this study, we used the YMC dataset to demonstrate a novel EMD application to improve the performance of existing periodicity detection methods. A recent study showed that combining different pattern detection approaches to analyze microarray datasets is a valuable strategy to identify novel candidate cyclic genes [36]. In the future, it would be interesting to gain further understanding of how different periodicity detection methods respond to the same dataset and how the methods perform on different datasets. Moreover, web tools such as SCEPTRANS [37] provide a valuable resource for biologists to analyze and view microarray time-series datasets. Integrating our EMD application with web data compendium will help facing challenges of time-series microarray analysis.

## Conclusions

In this paper, we present a novel method for identifying under-detected periodicity from time-series microarray data by using EMD. Our analysis demonstrated that periodicity of under-detected genes might be interfered with between decomposed IMF oscillations. By using a protein complex coexpression analysis, we revealed that 56 genes were newly determined as periodic in a yeast time-series microarray dataset. We also used EMD as a universal benchmark to assess the performance of periodicity detection. Based on the strength of EMD to further decipher the characteristics of the temporal structure of time-series microarray data, the approach can be used to improve existing periodicity detection methods.

## Methods

### Microarray data preprocessing

Raw data from the YMC dataset [29] was downloaded from the Gene Expression Omnibus [38], accession number GSE3431. The compiled dataset was presented in a 9335×36 matrix and contained the gene expression profiles of 9335 probesets at 36 time points. We normalized the matrix by using the MAS5 algorithm from the Bioconductor affy package [39]. According to criteria described in Tu et al., we classified probesets that had at least three present calls as expressed. Of the 9335 probesets, 7955 were expressed. Of these probesets, we used 6537 probesets or unique annotated ORFs for data analysis.

### Empirical mode decomposition

We used an R/Bioconductor-based [40] EMD package [41] to extract IMF oscillations from the YMC dataset. EMD was performed using the default settings of the software package.

### Periodicity detection

Identification of periodic signals and p-value of detection were determined by using algorithm described in Tu et al. The ∼300 min period of the oscillatory intrinsic mode function (IMF) was determined based on calculating autocorrelation function (ACF). To assess the p-value of the level of statistical significance, the 300-min autocorrelation of the oscillatory IMF was compared with autocorrelation expected from random (Gaussian distribution) data with no periodicity. The cutoff criterion of p-value is 0.05. We requested the C-based computer code of the ACF algorithm from authors of Tu et al. and rewrote the source code by using R. We integrated this algorithm with EMD to search the periodic patterns derived from all of the possible combinations of IMF. The R source code and instructions to identify the under-detected genes by using our EMD approach is available at http://metadb.bmes.nthu.edu.tw/emd_ymc/.

### Protein complex coexpression analysis

We collected 210 protein complexes from the MIPS database [31]. Based on Liu et al. [30], nine highly coexpressed protein complexes were selected for coexpression analysis. The size or subcomponents of a protein complex varied from 15 to 81 (Table 1). Coexpression between a gene pair in the same protein complex was evaluated by querying the adjusted correlation scores (ACSs) from the SPELL compendium [32]. All possible combinations of gene pairs in the same protein complex were queried. To determine whether a gene pair was coexpressed at a higher frequency greater than the frequency expected by chance, we calculated the statistical significance of coexpression by using a randomization test (n = 10^6^). A gene pair was classified as coexpressed if its queried ACS was ranked in at least the 95th percentile of a background distribution of randomly selected ACSs from the same protein complex.

### Gene ontology enrichment analysis

Functional categorization of gene clusters was analyzed using GO::TermFinder [42]. GO standardizes gene vocabulary or terms and gene product attributes [43]. GO includes three categories: biological process (BP), molecular function (MF), and cellular component (CC). The functional categorization of gene clusters was annotated using GO terms for BPs.

## Supporting Information

Figure S1 **Expression profiles of five single IMF probesets.**
(TIF)

Figure S2 **Visualization of networks of three highly coexpressed MIPS protein complexes associated with oxidative phosphorylation.**
(TIF)

Figure S3 **Distribution of the number of extracted IMFs for genes associated with ribosome biogenesis (GO:0042254).**
(TIF)

Figure S4 **Boxplots of the expression profiles for (A) non-periodic, (B) PP, and (C) ND probesets.** A periodicity analysis was performed using an algorithm described in [35].
(TIF)

Table S1 **Summary of three highly coexpressed MIPS protein complexes associated with oxidative phosphorylation.**
(XLS)

Table S2 **Functional categorization of all enriched GO terms (BP) for the OX, RB, RC and PP gene clusters.** Statistical significance of enrichment is presented by –log(p-value).
(XLS)

Table S3 **A complete list of 1469 under-detected probesets.**
(XLS)
