# Supplementary figures and images for: Identification of Under-Detected Periodicity in Time-Series Microarray Data by Using Empirical Mode Decomposition
Source: PLoS One. 2014 Nov 5;9(11):e111719. doi: 10.1371/journal.pone.0111719 (PMC4221108; doi:10.1371/journal.pone.0111719)

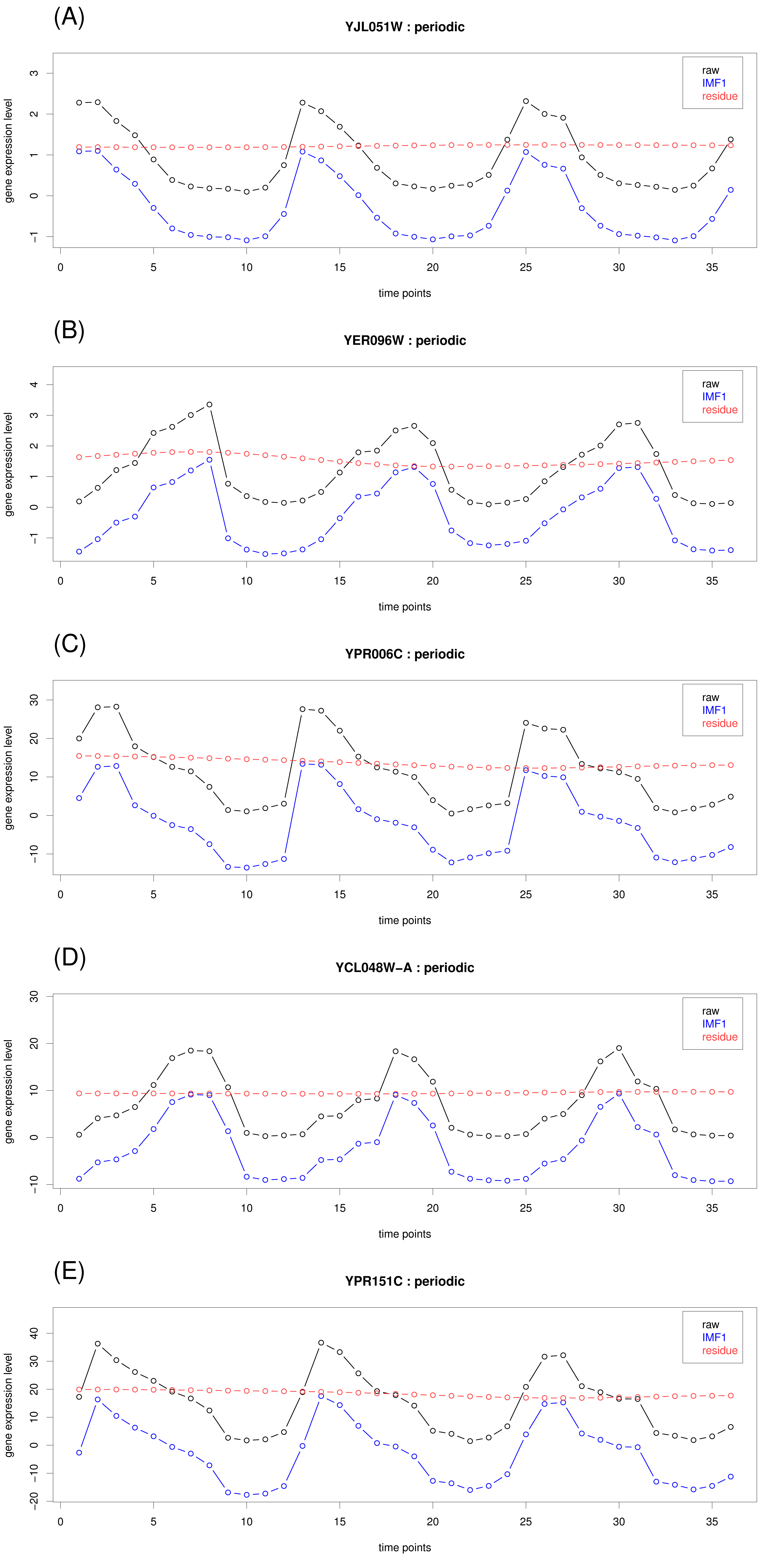

Supplement: Figure S1 — Expression profiles of five single IMF probesets. (TIF) [file pone.0111719.s001.tif]

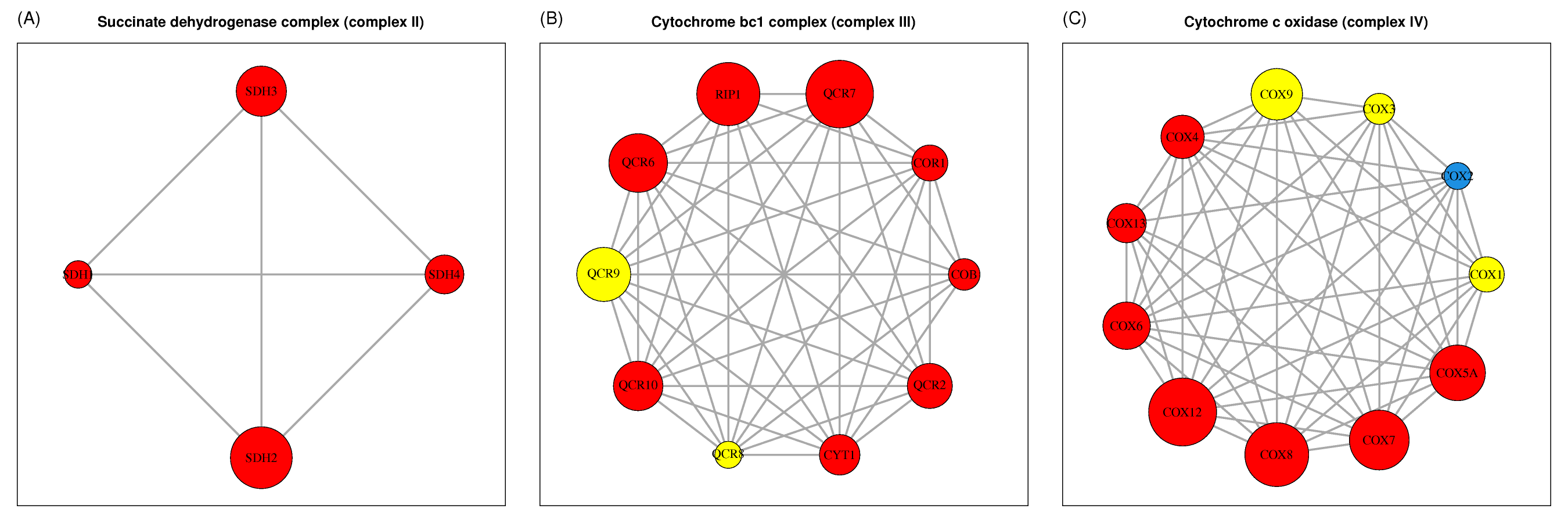

Supplement: Figure S2 — Visualization of networks of three highly coexpressed MIPS protein complexes associated with oxidative phosphorylation. (TIF) [file pone.0111719.s002.tif]

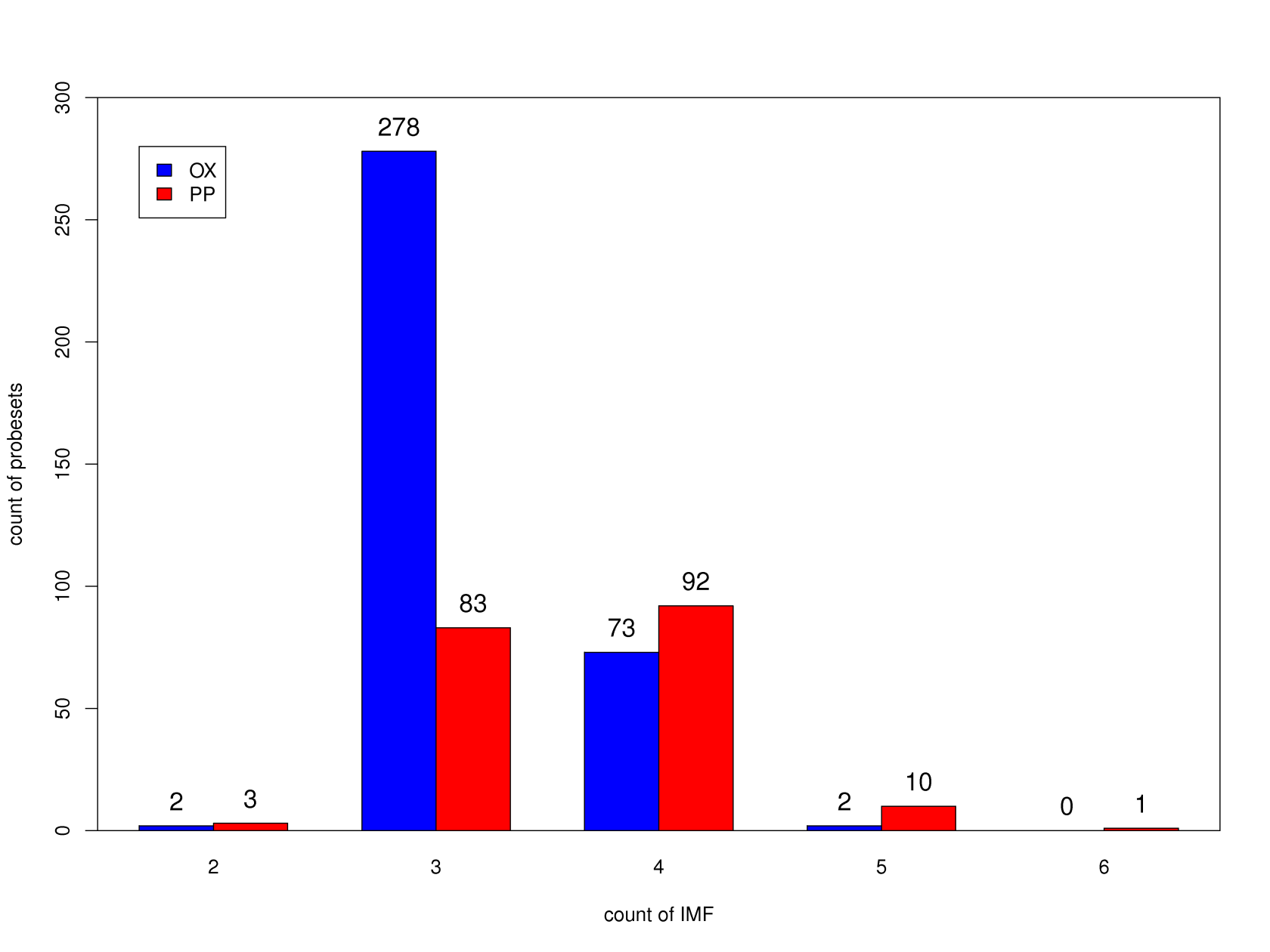

Supplement: Figure S3 — Distribution of the number of extracted IMFs for genes associated with ribosome biogenesis (GO:0042254). (TIF) [file pone.0111719.s003.tif]

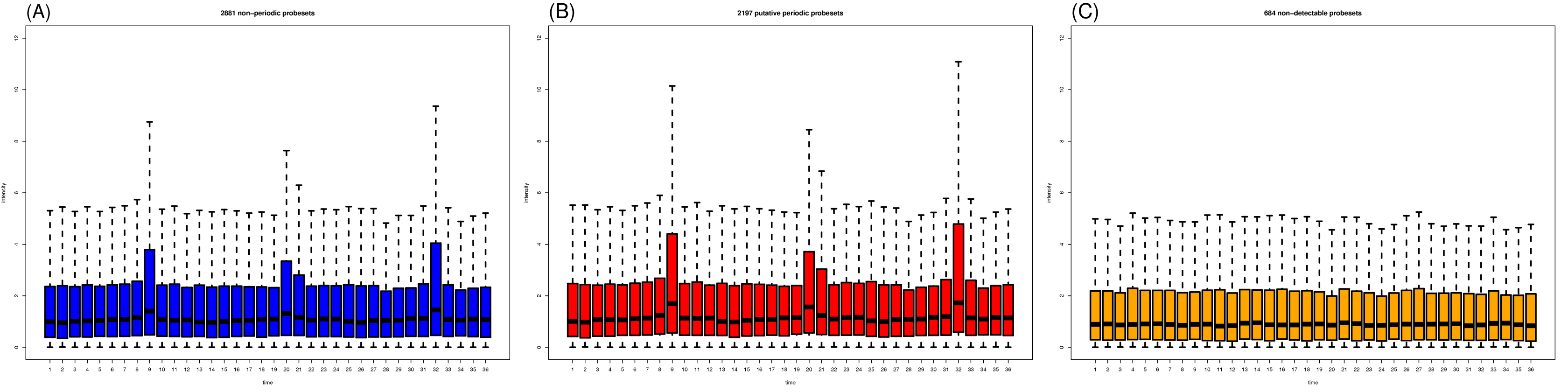

Supplement: Figure S4 — Boxplots of the expression profiles for (A) non-periodic, (B) PP, and (C) ND probesets. A periodicity analysis was performed using an algorithm described in [35]. (TIF) [file pone.0111719.s004.tif]
